# Supplementary material for: Are carbon emissions trading and green financial instruments synergistic? -Comprehensive quantitative research based on content analysis
Source: PLoS One. 2024 Mar 7;19(3):e0298601. doi: 10.1371/journal.pone.0298601 (PMC10919601; doi:10.1371/journal.pone.0298601)
Supplement: S1 Data — (ZIP) [file pone.0298601.s001.zip › Data/地级市Z_F工作报告.zip!/╒π╜¡/└÷╦«╩╨/└┤╫╘╣1⁄2╓┌║┼í╛┬φ┐╦╩2╛▌═°í┐_2018.docx]

各位代表：

　　现在，我代表丽水市人民政府向大会报告工作，请予审议，并请市政协委员和其他列席人员提出意见。

　　一、2017年工作回顾

　　2017年是本届政府任期的第一年，也是全市上下攻坚克难、奋发有为，推进“十三五”规划顺利实施，喜迎党的十九大胜利召开的一年。我们始终遵循习近平总书记“绿水青山就是金山银山，对丽水来说尤为如此”重要嘱托，着眼全省“两个高水平”建设大局，在省委、省政府和市委坚强领导下，切实推动“打好五张牌、培育新引擎、建设大花园”新定位、新要求及各项决策部署落地见效。生态环境状况指数连续14年全省第一，县级以上集中式饮用水源地、跨行政区域河流交接断面水质达标率均为100%，空气质量在全国74个排名城市中列第8位，市区空气质量指数(AQI)优良率达93.2%，PM2.5平均浓度33微克/立方米。经济运行稳中向好，实现地区生产总值1298.2亿元，增长6.8%，三次产业结构调整为7.7:42.9:49.4；一般公共预算收入112.91亿元，增长11.5%；规上工业增加值增长5.7%，服务业增加值增长7.7%，旅游产业增加值增长10%以上；固定资产投资增长11.5%，社会消费品零售总额增长11.3%。民生保障水平进一步提高，一般公共预算民生类支出299.37亿元，增长12.9%；城乡居民人均可支配收入分别为38996元和18072元，增长8.4%、9.8%，农民收入增幅连续9年全省第一；“五水共治”满意度、群众安全感满意率连续4年全省第一，食品安全满意度连续9年第一，生态环境质量公众满意度连续10年第一。

　　(一)坚持“尤为如此”的使命和担当，干好事关丽水长远发展的大事

　　创建浙江(丽水)绿色发展综合改革创新区从理念变为实践，成为加快高质量实现“两大历史使命”的总载体。我们紧紧围绕贯彻落实省第十四次党代会、市第四次党代会的战略部署，按照市委的战略构想，开展创新区总体方案编制工作。抓好深化细化具体化，组织制定创新区建设十大行动方案，谋划提出总投资超7267亿元的164个投资项目、17大项92小项改革任务，落实了工作推进责任清单。

　　办好首届世界丽水人大会等重大活动，为加快发展注入新动力。以“凝心聚丽、共建共享”为宗旨的首届世界丽水人大会，签约招商合作项目221个、总投资1358.7亿元。成功举办首届中国休闲度假大会，丽水摄影节暨第二届国际摄影研讨会、第26届全国摄影艺术展。

　　扎实推进地质灾害综合治理“大搬快治”，完成避让搬迁项目338个、搬迁25083人、已安置15075人，完成工程治理项目161个、解危7039人，核销地质灾害隐患点425处。打好全境剿灭劣V类水攻坚战，在全省率先完成全部2349个小微水体、1154个入河排污口整治任务，完成6座城镇污水处理厂一级A提标改造，实现“清三河”达标县全覆盖。以全国第四、全省第一的成绩创成全国文明城市，实现国家卫生县城全覆盖。

　　(二)增创生态保护新优势，“大花园”建设迈出坚实步伐

　　以最坚决的措施抓好中央和省两轮环保督察反映问题的整改，全年环境执法行政处罚案件770件、增长102.6%，罚款4088.06万元、增长145.1%，行政拘留31人、刑事拘留12人。实现一条红线管控重要生态空间，生态保护红线区域占全市国土面积比例从26.6%提高到31.9%。建立生态环境保护综合督查长效机制，否决不符合生态环保要求项目53个。协同制定《丽水市饮用水水源保护条例》，制定实施水气环境质量预测预警方案，在全省率先发布土壤污染防治工作方案。新增景宁、松阳2个国家生态县，云和、遂昌成为首批省级生态文明建设示范县。

　　“治城治乡”覆盖全域。丽阳坑水系沿线截污纳管全面完成，完成市区18个老旧小区、城中村雨污分流改造，实施提标改造管网177.2公里，新增城镇污水配套管网242.87公里、雨水管网157.6公里、供水管网97.03公里，新增城市道路37.6公里、绿道154.91公里，新竣工城市地下空间46.6万平方米，市区新增停车位5130个。完成城中村改造项目173个，拆除91.6万平方米。56个乡镇通过省级小城镇环境综合整治考核验收。完成城镇危旧房治理改造1478幢、农村危旧房改造20638户、困难家庭危房改造3430户。完成拆违1281.89万平方米、“三改”2065.09万平方米。农村生活垃圾分类处理覆盖36.5%的建制村。

　　“三美融合”成形成势。被评为全国“美丽山水城市”，完成“六边三化三美”整治线路76条、线长1216公里、沿线村庄116个，美丽公路建设10条250公里，美丽林相建设76.42万亩。缙云获评全国绿色发展与生态建设优秀城市，遂昌成为我市首个省级美丽乡村示范县，分别有81个村、198个村列入第四批中国传统村落、省首批传统村落。莲都堰头村、龙泉盖竹村、缙云仁岸村、青田洞背村，分别成为全国生态文化村、美丽乡村示范村、环境整治示范村、“十大最美乡村”。

　　(三)培育绿色发展新动能，产业转型升级取得明显成效

　　以拓市场为主导，“生态+”供给侧结构性改革成效凸显。全力推进农旅融合发展，全市新建农业景观带9条、休闲观光农业区(点)44个，莲都被评为全国休闲农业和乡村旅游示范县。新培育农产品旅游地商品生产经营主体234家、农产品转化为旅游地商品292个。新增“丽水山耕”品牌合作基地1122家，实现销售额41.79亿元。成功举办丽水山居·台湾民宿交流合作活动周系列活动，在全省率先实现“民宿保”全覆盖，实现农家乐民宿营业总收入31.23亿元。整治低小散企业716家，其中整合入园企业112家，淘汰落后产能涉及企业78家。制定实施生态工业“31576”五年行动计划，新增省级隐形冠军企业1家、培育企业11家，“浙江制造”品牌认证企业3家、重点培育企业16家，制造业与互联网融合示范试点企业8家，新增上云企业2094家，青田起步股份成为我市首家在上交所主板上市企业，浙江股权交易中心专门设立了“丽水生态经济板”。坚持全域旅游发展方向，入选第二批国家旅游业改革创新先行区，9县(市、区)均列入省全域旅游示范县创建名单。缙云仙都创5A基本具备验收条件，新增4A级景区1家，评定3A级景区村庄25家、A级景区村庄216家。

　　产业平台进一步夯实。全市开发区(园区)平均投资强度比上年增长12.1%，丽水开发区规上工业增加值增长8.8%。实现省级特色小镇县(市、区)全覆盖，完成特色小镇投资210.53亿元，其中特色产业投资占比70.4%。千峡湖旅游度假小镇投入试运营。侨乡进口商品城获评省十大小微企业集聚发展优秀平台。浙南茶叶市场连续3届入围中国商品市场百强。

　　产业后劲进一步增强。以总投资135亿元的万洋低碳智造小镇为代表，一批产业项目成功落地。新引进大项目108个，全年实际利用内资360.81亿元、外资2.17亿美元。规上工业战略性新兴产业、高新技术产业、装备制造业增加值分别增长4.2%、13.7%、12.8%，新产品产值率36.6%。政府产业基金到位资金10.29亿元，撬动社会资本43.15亿元。7个县(市)入围阿里研究院公布的“电商百佳县”，为全国入围数量最多的地级市。

　　(四)探索“两山”转化新通道，形成改革撬动、创新驱动、开放推动、项目带动良好态势

　　“最多跑一次”改革聚变效应不断显现。市本级“最多跑一次”事项达到829项，县(市、区)平均达到698项，全市“最多跑一次”实现率97.2%，群众满意率列全省第二。变“跑多门”为“跑一门”，市、区两级11个政务服务场所合而为一。变“群众跑”为“数据跑”，所有投资项目实现网上审批、核准、备案，在全省率先探索中介超市“网竞平台”、推行“电子图审”。借力“最多跑一次”改革，推动全市公共资源交易平台整合到位，实现国地税大厅合并县(市、区)全覆盖。联动推进农村产权、金融、扶贫改革，首创农房流转使用权抵押贷款模式，“三权”抵押贷款余额超百亿。完成五大国有公司重组整合。开通水电产权(股权)流转平台。新增11个国家级改革试点、47个省级改革试点。

　　创新驱动持续加力。绿谷信息产业园成为省级科技企业孵化器，园区主营业务收入和税收分别增长31.7%、68.1%。新增省级科技创新服务平台1家、企业研究院5家、高新技术企业研发中心17家、众创空间3家，国家高新技术企业45家、省级科技型企业195家。新获中国专利优秀奖1项，专利申请量和授权量分别增长37%、15.2%。成功举办2017中国“互联网+公共服务”创新大会，腾讯“互联网+”创新基地落户丽水。庆元李玉院士专家工作站获评“全国示范院士专家工作站”，首个外籍院士工作站落户古堰画乡特色小镇。

　　市域开放程度进一步提升。主动对接和参与“一带一路”、长江经济带和海西经济区建设，加快建设青田华侨经济文化试验区，庆元成功创建国家级铅笔出口质量安全示范区，丽水无水港投入运营。建成侨乡投资项目交易中心。着力打造山海协作工程升级版，青田与平湖共建“飞地”产业园。扎实推进对口支援工作，与四川6个县(区)建立扶贫协作结对关系。以“赶街”模式为代表的电商服务，拓展至全国23个省(市、自治区)的50个县。

　　项目建设加快推进。省、市重点建设项目分别完成年度投资计划的142.2%、123.1%，37个PPP项目签订合同并开工建设。大干快上重大交通项目，金丽温高速丽水北互通、庆元高速连接线等项目顺利建成，衢丽铁路“单线改双线”等项目加快推进。实施水利“双百亿”工程，单体百亿级投资项目缙云抽水蓄能电站正式开工。金丽温输气管道工程丽水市区、缙云县建成通气。

　　(五)推进民生福祉新改善，奏响以人民为中心发展主旋律

　　统筹抓好增收就业社保。城乡居民人均可支配收入增长快于经济增长，低收入农户人均可支配收入增长16.5%。完成全市商业保险助推扶贫工作“1+6”新体系构建，支出型困难家庭纳入低保。消除集体经济薄弱村855个，完成省定三年目标的77.2%。“超市、电子商务、农家乐民宿创业培训项目”获评全国创业就业服务优秀项目，新增城镇就业18386人。深入推进全民参保计划，户籍人口基本养老保险参保率、基本医疗保险参保率分别达84.5%、98.8%。

　　加快提升教育、医疗等民生之要。龙泉、云和、景宁创成省教育基本现代化县，市县两级全面建立政府领导兼任特邀督学制度，普通高校录取率为93.33%，丽水学院列入省硕士点立项建设单位，丽水职业技术学院列入省优质高职院校建设单位；建立市级医联体6个，乡镇卫生院业务用房建设达标率与设备配置率均达95%以上。扎实推进住有所居、老有所养、残有所助、困境儿童有保障工作，交付保障性安居工程5281套；创成首个省级“老年友好城市”，新增城乡社区居家养老服务照料中心246个、养老机构床位1362张，实现80岁以上老年人意外伤害保险全覆盖；市残疾人托养中心投入运行，残联康复医院挂牌营业，残疾人社会保险补贴实现全覆盖；困境儿童享受生活保障6357人。推进文化惠民，市科技馆、非遗馆建成开放，“乡村春晚”入选国家文化蓝皮书，龙泉成为国家历史文化名城、全省文化产业十强县。成功举办第四届市运会，实现省体育强县全覆盖。实现建制村客车“村村通”，城乡公交覆盖市区所有乡镇，电动汽车分时租赁网络覆盖8个县(市、区)。民族宗教工作和妇女儿童、慈善、红十字、移民、档案、史志等事业取得新成效。

　　积极打造“全国最平安城市”。继夺取“平安金鼎”后，又获评全国综治优秀市。成为“雪亮工程”重点支持城市，实现“基层治理四平台”建设全覆盖，庆元“五台合一”获批国家级标准化试点。强化安全生产，各类安全生产事故起数、死亡人数连续14年“零增长”。强化公共安全，6个县(市、区)创成省级农产品质量安全追溯体系县，新建扩改建避灾安置点258个。强化社会治安综合治理，刑事案件、治安案件分别下降28.1%、16.6%。国防动员和后备力量建设取得新进展。

　　各位代表！过去的一年，我们始终坚持党的领导，加快转变政府职能，扎实推进政风建设。主动接受人大法律监督、工作监督和政协民主监督，坚持重大事项向人大报告、政协通报制度，办结人大代表建议279件、政协提案370件。完善并出台《丽水市人民政府重大行政决策程序规则》，市行政复议局挂牌成立。致力于提升政府执行力和公信力，对30项重点工作、30个重点项目实行“每周一报”清单管理。把构建亲清政商关系与精准服务企业紧密结合起来，办结企业反映问题1459个、办结率88.4%。严格落实政府党组主体责任，锲而不舍抓好中央八项规定精神和省委、市委相关规定落实，坚决整治“四风”，“三公”经费支出下降16.4%。

　　各位代表！过去一年的成绩来之不易，这是市委正确领导的结果，是全市人民团结拼搏的结果。在此，我代表市人民政府，向全市人民，向人大代表、政协委员，各民主党派、工商联、人民团体和社会各界人士，向驻丽部队官兵和省部属驻丽各单位，向关心和支持丽水发展的海内外朋友们，表示衷心感谢！

　　我们也清醒看到，经济社会发展中还存在不少矛盾和问题。主要是：“绿水青山”转化为“金山银山”的通道还没有充分打开，生态经济新动能、新增量的培育亟须加速，地区生产总值增速未达预期目标；生态环保基础设施有欠账，城市建设截污纳管任务重；中心城市集聚辐射带动能力有待提升；群众可持续增收难度增大，教育、医疗等民生领域与“两个高水平”要求比，还有不少短板；政府机关干部创造力尚未充分激发，少数部门和工作人员的服务意识、争先意识、担当意识不够强，个别民生实事落实不到位。我们一定高度重视这些问题，采取更加有力措施，切实加以解决。

　　二、2018年主要目标任务

　　争先进位大赶超，是当前丽水发展与中国特色社会主义新时代同频共振的时代音符。建设生态文明在新时代已提升为“千年大计”，对于有着“中国生态第一市”美誉的丽水来说，惟有审时度势、因势利导，才能赢得新时代的新发展。面对互联网时代、高铁时代、消费升级时代，我们的生态环境优势不断凸显，区位交通条件不断优化，正在迎来绿色发展“蝶变期”！这也是我们后发赶超，进入高质量发展快车道的重要关头。“时来易失，赴机在速”，决不可无视机遇，更不能错失机遇！

　　2018年是全面贯彻党的十九大精神的开局之年，是改革开放40周年，是决胜全面建成小康社会、实施“十三五”规划承上启下的关键一年。做好今年工作，必须牢牢把握高质量发展“时间窗口”，把“不进则退、小进也是退、慢进更是退”的区域竞争压力转化为动力，坚持好字当头、快字为要，明确目标引领赶超，树立标杆激励赶超，突出重点精准赶超，改进作风促进赶超，严明纪律保障赶超；必须坚持以绿色为主基调、发展为主旋律，拉高标杆创一流，扬长补短创优势，擦亮生态底色、做强发展底子，努力成为全省发展新引擎和新的经济增长点，高起点打造“两山”理念示范区，加快实现绿色崛起。

　　今年政府工作的总体要求是：高举习近平新时代中国特色社会主义思想伟大旗帜，全面贯彻党的十九大、省第十四次党代会、市第四次党代会和中央、省委、市委经济工作会议精神，始终遵循“尤为如此”重要嘱托，坚持高质量发展根本要求，坚持以“八八战略”为总纲、以稳中求进为工作总基调、以供给侧结构性改革为主线，明目标、树标杆、争先进位大赶超，全力打好五张牌、培育新引擎、建设大花园，全面推进浙江(丽水)绿色发展综合改革创新区建设，为加快高质量实现“两大历史使命”奠定坚实基础，奋力开辟“绿水青山就是金山银山”新境界。

　　综合分析发展大势与丽水实际，建议经济社会发展主要预期目标为：地区生产总值增长7%，一般公共预算收入增长与经济增长基本同步；生态环境质量持续提升，并继续保持全省第一、全国领先；城乡居民收入增幅高于经济增幅，走在全省前列，农民收入增幅力争继续保持全省第一；研究与试验发展(R&D)经费支出增幅比地区生产总值增幅高10个百分点以上；固定资产投资增长11.5%以上；单位生产总值能耗、节能减排降碳等约束性指标均按省定目标要求严格执行，空气质量指数(AQI)优良率达到90%以上，地表水I-III类水比例占98%以上。

　　(一)狠抓创新区建设，在落实落地落细落小上下功夫

　　坚持生态保护第一。以全市全员全时全程的生态自觉、生态自律，切实打造最优绿色生态涵养区。强化“多规合一”促保护，坚持全域统筹、生态引领、城乡一体，科学划定生态保护、永久基本农田保护、城镇开发边界，加快实现空间规划“一张图”。强化顶格标准促保护，坚持地上地下、各行各业都要高标准，统筹制定涵盖一二三产的产业准入负面清单，统筹抓好全方位立体化自然生态保护与修复。强化责任落实促保护，启动市级环保督查，探索建立环保信用评价体系，编制市级自然资源资产负债表，全面开展领导干部自然资源资产离任审计。强化共建共享促保护，启动国家生态文明建设示范市创建，推进美丽乡村、美丽田园、美丽河湖、美丽林相一体化创建。创建省级美丽乡村示范县1个、示范乡镇10个、特色精品村28个，打造美丽乡村风景线10条。推动田园综合体创建工作县(市、区)全覆盖，新增休闲观光农业区(点)27个。系统推进“一江丝路盛景、十城秀美河川、百里滨水画卷、千村碧水映绕”水利项目建设，完善河长制，实施湖长制，完成河湖库塘清淤100万方、河道综合整治70公里。建设美丽林相65.57万亩，种植珍贵树360万株。

　　强化综合交通先导。提速实施综合交通建设“168”工程，加快对接全省大通道、融入全省大格局，构建连接省、市、县、乡域“1小时交通圈”。加快建设衢宁、金台铁路，开工建设衢丽铁路，积极谋划和推进丽水接轨杭义温高铁连接线、丽水至云和铁路、温武铁路。确保丽水机场开工建设，统筹抓好市域通用机场规划与前期。开工建设景文高速，加快谋划金松(遂)龙高速。加快水东综合客运枢纽、瓯江航道建设。抓好浙西南景区化高速公路规划对接，扎实推进通景公路建设。加快330国道温溪至船寮大过境项目前期。建设“四好农村路”，完成农村公路提升改造等项目1350公里。统筹推进基础设施网络化、现代化，加快实施天然气“县县通”工程，加快推进500千伏丽西输变电工程前期和西南电网补强工程，加快完善电动汽车充电设施网络，新增充电桩500个。

　　突出平台建设带动。围绕深入打造“美丽浙江”大花园最美核心园，以构建绿色发展高地、健康养生福地、最佳旅游目的地为目标，聚焦国家公园创建区、休闲养生新区、千峡湖生态文化旅游区、松遂“诗画田园”样板区、生态适宜型产业示范区等五大平台，谋深谋细，抓实抓好。尤其要通过高起点创建国家公园，打造国际生物多样性保护研究平台，统筹和盘活全域生态资源，促进形成“国家公园+美丽城市+美丽乡村+美丽田园”的市域空间形态，促进生态优势转化为竞争优势、发展优势。谋划建设沿瓯江文创产业带，大力发展“丽水三宝”历史经典产业，实现文化产业增加值增长12%左右。推进特色小镇“产、城、人、文”融合发展，完成特色小镇年度投资150亿元以上，努力使特色小镇成为创业创新集聚地、产业发展动力源。深度激活侨乡优势资源，深入推进侨乡投资项目交易中心建设，打造省级大平台。以年内“厅市共建”平台全覆盖为努力方向，进一步争取省直单位对创新区建设的关心和支持。

　　切实抓好有效投资。坚持选商引资“一号工程”不动摇，用好一把手“五亲招商法”，强化争的意识、抢的劲头、引的力度，持续掀起大招商、招大商热潮。强化区域统筹招商和专业化招商，突出以商引商、产业链招商。扎实推进央企、浙商、外资、民资、侨资“五资齐抓”，引进大项目100个，确保实际利用内资345亿元、外资2亿美元，浙商回归到位资金200亿元。着眼于培育植入式发展新引擎，统筹编制全市重大产业项目谋划盯引清单，签约总投资20亿元以上重大产业项目30个以上，重大产业项目落地率达到50%以上。突出抓好市县长项目工程谋划与落地，市长、副市长共谋划50亿元以上大项目好项目不少于4个，县(市、区)各谋划14亿元以上项目不少于3个，落地率达到50%以上。分层分类分级梳理创新区建设年度项目清单、责任清单，确保交通基础设施投资增长10%以上，高新技术产业投资、生态环境和公共设施投资、民间投资分别增长15%以上。实行首届世界丽水人大会签约项目、市区三产服务业项目落地责任清单管理，确保项目履约率、开工率、到资率、投产率同步提升。

　　(二)以“最多跑一次”改革为牵引，努力提高改革开放水平

　　扎实推进“最多跑一次”改革，积极推动重点领域改革。确保“最多跑一次”改革标准化全覆盖、行政权力事项全覆盖、市县乡村四级全覆盖，加快实现“一证通办”“全域通办”。建好“一网集成、信息共享”公共服务数据平台，并与“基层治理四平台”有效衔接，年底前所有民生事项和企业事项实现“一次办结”，其中80%以上开通网上办理，50%以上的民生事项实现“一证通办”。推进企业投资项目专业代办服务全覆盖，全面实现企业投资项目开工前审批全流程最多跑一次、最多100天。聚力打造最优投资环境，全面实施“区域能评、环评+区块能耗、环境标准”制度，推进企业投资项目承诺制改革、商事登记“证照分离”改革试点，推动统一社会信用代码改革落地，构建全市统一公共资源交易监管体系，实现综合行政执法派驻乡镇(街道)、功能区全覆盖。积极推进生态产品价值实现机制、农村产权和金融、水电产权、碳汇交易、“三块地”等重大改革试点，抓好创新区重大改革行动“一县两试点”，加快旅游“三化”改革，深化国资营运公司市场化改革。

　　扎实推进供给侧结构性改革。围绕增加高效优质供给，制定重点产业导向清单，切实抓好“亩均论英雄”的要素市场化配置改革，推行企业对标竞价的“标准地”制度，新批工业用地的30%按照“标准地”制度供地。严格执行环保、能耗、技术、质量、安全等法规标准，有效处置“僵尸企业”，淘汰50家企业落后产能，促进300家以上低小散企业完成整治任务。深化“精准服务企业、振兴实体经济”专项行动，减轻企业负担30亿元以上。

　　扎实推进开放强市。深度融入“一带一路”、长三角、海西区和全省大湾区大花园大通道大都市区建设，主动对接上海自贸区、浙江自贸区等重大区域改革，办好闽浙赣皖九方经济区联席会议，不断深化与宁波、嘉兴、湖州等山海协作结对市全面合作，着力打造“飞地”经济合作共享新模式。积极推进跨境电子商务产业园建设，抓好云和国家外贸转型升级基地和龙泉外贸公共服务平台建设。深入推进华侨要素回流，把华侨经济文化试验区打造为全市海外华人华侨集聚发展新平台，把侨乡进口商品城打造为华东乃至全国的进口商品批发集散中心。积极推动“青瓷之路开放之路”重现辉煌，继续做好龙泉窑大窑-金村遗址“海上丝绸之路”申遗工作，支持龙泉策划举办龙泉青瓷“一带一路”沿线国家重要城市、联合国大厦巡展。完善国际友城布局，打造线上线下“世界丽水人之家”。

　　(三)全面推进绿色发展，加快建设现代化生态产业体系

　　以“丽水山耕”打造农业版“浙江制造”为契机，推动生态精品农业提质增效。强化“丽水山耕”国际认证联盟第三方认证，加快构建国内领先、比肩国际的“丽水山耕”农业区域公共品牌认证标准体系和溯源管理体系，实现省级农产品质量安全追溯体系县(市、区)全覆盖。建成30万亩海拔600米以上“丽水山耕”绿色有机农林产品基地，实现省内高速服务区销售点全覆盖，品牌农产品年销售额60亿元。建成“丽水山耕”全产业链创新服务综合体一期工程。抓好现代农业产业统计制度创新国家试点，创建国家农业高新技术产业示范区、浙江(丽水)农业绿色发展先行示范区。开展县域无公害农产品产地整体认定，大力发展历史经典农业。

　　以争创省级生态工业试点市为推动，加快生态工业第一经济高质量发展。深入实施“丽水生态制造2025”和生态工业“31576”五年行动计划，加快传统制造业改造提升和新动能培育，规上工业增加值增长7%以上，工业投资增长15%以上，重点技改投资增长15%以上。强化高端装备制造、新材料、生态能源及生物医药三大千亿级产业培育，加快缙云机床小镇、龙泉汽车空调产业创新服务综合体建设，打造特钢新材产业基地，推动建设钛材料研究院、钛产业园区和钛谷小镇，积极推进开发区维康大健康产业园、方正电机产业园、中广电器产业园等重大项目。强化产业集群生态化发展，扎实推进松阳茶叶加工业省级传统制造业改造提升试点，以及庆元竹木加工、松阳不锈钢、龙泉汽车空调零部件、云和木制玩具、开发区合成革、青田制鞋业等6个省级“机器换人”分行业试点。强化园区平台，发挥好丽水开发区生态工业主平台作用，开发区工业企业亩均税收和亩均销售收入分别提高12%、15%。积极支持丽景园、丽缙园建设发展，大力推进万洋低碳智造小镇等产业园项目，新增6家以上小微企业园区，新建标准厂房30万平方米，推动300家以上企业进入园区集聚转型发展。强化企业梯度培育，培育重点拟上市企业8家，列入省级隐形冠军培育企业5家以上，年产值10亿元骨干龙头企业达到20家以上。强化创新驱动，深入实施高新技术企业和科技型企业“双倍增”计划，新增高新技术企业45家、科技型中小微企业150家，全面推广工业科技特派员制度，确保高质量通过国家知识产权试点城市验收。

　　以打造“诗画浙江”的鲜活样板和世界一流生态旅游目的地为目标，做大做强生态旅游第一战略支柱产业。切实抓好国家全域旅游示范区、旅游业改革创新先行区建设，打造市域统一品牌，推进景区串点成线，加快实现旅游发展国际化、标准化、全景化。新建、续建旅游项目300个以上，旅游投资增长20%以上。加快缙云仙都、古堰画乡、云和梯田、遂昌金矿5A级景区创建，确保缙云仙都创成5A级景区，新增2家4A级景区。推进“旅游+”产业融合发展，新创建市级及以上“旅游+”示范区或基地30个，重点培育20个农旅融合集聚区。积极培育高端旅游酒店。依托浙皖闽赣国家生态旅游协作区、长三角高铁旅游联盟、金丽温衢旅游联合体、长寿之乡绿色产业联盟等区域合作平台，抓好旅游精准营销，延长游客停留天数，提高旅游消费水平。大力培育旅游“名导名嘴”，解决好旅游紧缺人才问题。

　　着力提高新经济、服务型经济发展水平。积极培育新兴业态，发展平台经济、分享经济、体验经济、创意经济，壮大总部经济。打造“三园一镇”新经济主阵地，杭州丽水海创园建成并投入使用，实施省级软件和信息服务产业特色基地重点示范项目30个以上，数字经济核心产业增加值增长12%左右，新增上云企业2000家。深化与中国信息化百人会战略合作，积极争取数字生态经济研究院落户丽水。推动军民融合产业发展，支持缙云建设省级军民融合创新示范区。扎实推进全市11个重点服务业集聚区建设，服务业重点行业项目完成投资65亿元。加快创建国家农村电商示范区，推动“赶街”等电商模式拓展升级。研究产业政策，助推绿色能源、森林康养等产业发展，推动中医药产业化。倡导绿色消费，创建放心消费城市，推动生活性服务业向精细化、高品质转变。

　　(四)大力实施乡村振兴战略，加快城乡融合发展

　　全面落实“产业兴旺、生态宜居、乡风文明、治理有效、生活富裕”总要求，打造乡村振兴示范区。以规划先行引领乡村振兴，通过规划“一张图”优化城乡布局，集成政策、资源优势，因村制宜、分类指导。以活态保护促进乡村振兴，制定并落实乡村发展“负面清单”，推进松阳全国传统村落保护利用试验区和“拯救老屋”整县推进试点建设，推动传统村落保护与自然生态保护相得益彰。以产业融合支撑乡村振兴，在落实粮食安全市县长责任制的同时，集成“丽水山耕”“丽水山居”品牌优势，不断丰富创新农村一二三产业融合发展模式，创建美丽经济示范项目20个、A级以上景区村庄200个，积极探索利用农民闲置住房发展乡村旅游、养老等产业的有效方式，促进农民增收。以改革创新激活乡村振兴，加快推进生产、供销、信用“三位一体”农合联改革，联动推进农村产权、金融、扶贫改革，申报金融支持乡村振兴改革试验区，全面完成农村土地确权登记颁证工作，推进全域土地综合整治，促进农村资源变资产、资产变资金。以主体更新助力乡村振兴，大力培育新型职业农民，推进创客入乡、市民下乡、企业兴乡、能人进乡、乡贤回乡，推动公共服务和要素下乡。以文明善治保障乡村振兴，全面开展移风易俗行动，继续实施“信贷惠德”行动，弘扬耕读文化、慈孝文化，新建农村文化礼堂248个；打造新时代“枫桥经验”升级版，积极推进“基层治理四平台”向村级延伸，创建自治、法治、德治“三治”结合的善治示范村。

　　强化中心城市能级，提升城乡融合发展牵引力。按照国际名城、绿色城市要求，高起点构建生态品质之城规划新体系，深化控规修编，深度谋划中闲区块发展规划，加快实现城市设计全覆盖。着力提升城市商贸服务功能，加快现代商贸综合体、特色商业街区等市场主体建设和培育，加快推进江滨商务中心和处州府城、南明湖国际休闲养生港等一批城市休闲养生项目建设。着力提升城市基础设施水平，开展海绵城市示范项目创建，实施城东区块地下综合管廊PPP项目，全市建成、改造污水配套管网100公里，新建绿道140公里，开工建设滩坑引水工程，谋划城市轨道交通，继续推进市区桐岭路等重大项目建设。着力提升城市品位，与城市有机更新、撤村建居相结合，完成市区城中村改造120万平方米；制定城市景观立面改造导则，启动中山街立面改造。向产城融合、人才集聚、精细管理要城市竞争力，加快产城融合项目招商引资，进一步研究完善“人才强市”政策体系，实施“丽水籍人才智力回归”工程，深化院校人才智力项目合作，打造人才生态最优市，建立巩固全国文明城市创建成果长效机制，明晰管理职责，完善管理导则，推进城市管理标准化、精细化、数字化。围绕“一县一主题、一县一特色”，加快打造“产居游共融、人景城共享”的美丽县城和城镇集群，年内完成70个以上乡镇(街道)小城镇环境综合整治任务，继续深入推进“三改一拆”和“无违建县(市、区)”创建。

　　(五)坚决打好“三大攻坚战”，为丽水发展创造良好环境

　　未雨绸缪打好金融风险防控攻坚战。坚决守住不发生区域性金融风险的底线，加强不良资产处置和“两链”风险化解，加强互联网金融风险整治，严厉打击违法违规金融活动，年底不良贷款率下降到1.5%以内。着力增强金融服务实体经济能力，加快筹建民营银行，加强政银企、政银保合作，发挥好政策性融资担保公司作用，深化绿色金融供给改革。

　　精准施策打好低收入百姓增收攻坚战。实施低收入农户全面小康计划，坚持一村一策、一户一策，确保不落下一户、不落下一人。动态消除家庭人均年收入4600元以下贫困现象，巩固脱贫成果。按照兜底线、织密网、建机制的要求，加大社会救助力度，实施低收入农户补充医疗保险制度，努力解决因病致贫问题。加大“一亩山万元钱”林下经济、丽水中蜂“十箱万元助低收入农户增收”、开发村(社区)公益性岗位、光伏小康等精准扶贫项目实施力度，推动低收入农户收入增幅达到两位数、高于全市农民收入平均增速。深入推进搬迁扶贫，完成异地搬迁8000人。落实股份经济经营体制创新试点村500个，新建集体经济物业项目140个，全面消除集体经济薄弱村。

　　问题导向打好污染防治攻坚战。以开展第二次全国污染源普查为契机，彻底摸清污染源底数，精准落实污染源治理和监管。打好碧水保卫战，建立消除劣V类水体长效机制，落实流域上下游水污染联防联控机制。新建、扩建、提升污水处理厂7座，加快推进并确保腊口污水处理厂于2020年建成，启动水阁污水处理厂二期项目。以力争2019年基本完成、2020年完善升级为目标，推进所有城镇和开发区(园区)截污纳管全覆盖。全面推进“污水零直排区”创建工作，年内所有省级以上开发区(园区)创成“污水零直排区”。打好蓝天保卫战，推进城市浊气、工业废气、农业废气、汽车尾气、燃煤烟气“五气共治”，完成挥发性有机物排放企业治理任务，推进油烟污染餐饮单位专项整治到位。打好净土保卫战，强化土壤环境监测网络建设和污染防治，确保土地污染面“零增长”、污染地块安全利用率不低于91%。出台最严格的农药和化肥区域准入标准，实现化肥农药使用强度、使用总量“双下降”，降低农业面源污染。推进有害固废、生活固废、污泥固废、建筑固废、再生固废“五废共治”，完成丽水市工业固体废物综合处置场等固废处置设施建设，确保丽水市餐厨废弃物处置中心建成运营。扎实推进城乡生活垃圾分类处理，启动各县(市)垃圾焚烧发电项目和餐厨废弃物处置项目建设，城市和农村生活垃圾分类收集覆盖面分别达到80%以上、50%以上，城镇生活垃圾回收利用率、资源化利用率、无害化处理率分别达30%以上、30%以上、100%，农村生活垃圾回收利用率、资源化利用率、无害化处理率分别达30%以上、80%以上、99%以上。

　　(六)突出富民惠民安民，努力给人民群众带来更多的获得感、幸福感、安全感

　　着力做好“富民”文章。千方百计促进城乡居民增收，打好促农增收组合拳，适时适度提高最低工资标准，健全职工工资水平决定和正常增长机制。强化高校毕业生、就业困难人员、农村转移劳动力、退役士兵等重点人群就业创业服务，举办职业技能培训班300个、投入培训补贴资金1500万元、培训2万人，新增城镇就业1.4万人，实现城镇失业人员再就业5000人，城镇登记失业率控制在3.5%以内。继续抓好来料加工，年发放加工费20亿元以上。推进企业人才集合年金计划，稳步提高退休人员基本养老金和城乡居民基础养老金标准，全面实现低保标准城乡一体化。

　　着力做好“惠民”文章。坚持教育优先发展，持续推动莲都区基础教育“三年攻坚”，推动遂昌、庆元创成省教育基本现代化县，支持相关县(市)创建全国义务教育优质均衡发展县、实施省基础教育重点县提升工程。完善优秀教师激励机制，实现中小学教师“县管校聘”管理改革县(市、区)全覆盖。实施第三轮学前教育行动计划，支持庆元开展农村学前教育补短板改革试点。优化教育资源供给，减少义务教育阶段学校超标准班额班级253个，确保小学、初中起始年级不出现超标准班额现象，到2019年与全省同步实现全面消除小学、初中大班额。支持丽水学院、丽水职业技术学院提高办学水平，服务绿色发展。全面推进“健康丽水2030”行动，提高全方位全周期保障人民群众健康水平。完善“双下沉、两提升”长效机制，推进缙云县域医共体建设省级试点，实施偏远山区“医疗服务全覆盖”精准对接计划。深化医养护一体化改革，80%以上的医疗机构开设老年人绿色通道，开展医养结合服务的乡镇卫生院(社区卫生服务中心)，每个县(市、区)不少于3所。办好丽水半程马拉松赛等大型赛事，重视老年人体育，实施100个小康体育村升级工程，新增体育场地面积10万平方米。弘扬社会主义核心价值观，系统研究提升瓯江文化品牌，实施“处州文献集成编撰工程”。持续推进“文化三下乡”，创成“乡村春晚”国家公共文化服务体系示范项目。加快瓯江文苑特色民办博物馆群建设。建成一批街头书吧、“城市书房”。推进媒体深度融合。切实抓好二轮修志。支持景宁承办全国民族自治县全面建成小康社会经验交流现场会，推进景宁民族地区城乡融合发展改革创新试点。完善社会救助、社会福利、公益慈善事业、优抚安置制度，重视人口与计划生育工作，加强妇女儿童权益保护，加强残疾预防和康复服务。坚持房子是用来住的、不是用来炒的定位，加快建立多主体供给、多渠道保障、租购并举的住房制度，加大商住用地供应力度，创新房屋征收安置政策，促进房地产市场平稳健康发展。抓好“厕所革命”等民生关键小事，按照生态、洁净、无臭和节约、实用的要求，新建或改造提升城区公厕35座、景区厕所106座，改造农村厕所4650座。侯卫东官场笔记

　　着力做好“安民”文章。出台《平安丽水2025行动纲要》，制定推进城市安全发展实施方案，完善贯穿各个领域、贯通各个层级的应急指挥体系，加强应急处置演练，提升应急处置能力。坚决打赢大搬快治“清零战”，进一步摸清地质灾害隐患底数，加强动态监测，即查即防即治，确保实现已知地质灾害隐患“解危清零”。开展城镇危房治理情况“回头看”，完成农村危房治理改造21022户。深入实施安全生产综合治理三年行动，坚决遏制重特大安全事故。加强防汛防台、森林消防等工作。开展扫黑除恶专项斗争和农村基层作风巡查，依法打击和惩治黄赌毒黑拐骗等违法犯罪活动。开展“传承红色基因、汇聚强军力量”主题国防教育，支持国防后备力量和驻丽部队建设，加强退役军人服务保障工作，强化国防动员能力。

　　各位代表！新的一年，按照民生实事群众提、大家定、政府办的理念，市政府提出了加快截污纳管、实施地质灾害隐患“解危清零”行动、推进交通便民、优化基础教育资源供给、完善全民医保、加强医养结合的养老服务体系建设、深化助残服务、实施精准扶贫项目、加强食品安全监管、推进农村危房治理改造、推进“厕所革命”、加快推进天然气管网建设和入户工程、实施农村饮用水安全巩固提升工程等候选项目。经本次人代会讨论确定后，我们会以“钉钉子”精神，办好每一件实事。

　　我们还将着眼于解决发展不平衡不充分问题，全力打好综合交通提升、国家公园创建、政策处理安置、“丽水山耕”“丽水山居”品牌提升、美丽乡村美丽田园美丽河湖美丽林相一体化创建、生态工业提质增效、城市环境大提升、创建国家农村电商示范区、基础教育和基层医疗卫生资源优化供给、打造沿瓯江文创产业带十大会战，并梳理解决一批历史遗留问题。

　　三、努力建设人民满意政府

　　新时代、新使命，对政府自身建设提出了更高要求。我们将自觉维护以习近平同志为核心的党中央权威和集中统一领导，深入学习贯彻党的十九大精神，认真开展“不忘初心、牢记使命”主题教育，以人民满意为标准，以“两强三提高”为导向，不断提升政府施政能力和服务水平。

　　打造学习型政府。坚持以习近平新时代中国特色社会主义思想武装头脑、指导实践，在学懂弄通做实上下功夫，树立“四个意识”、坚定“四个自信”，提高政治站位、找准工作定位。大力推进全员学习、终身学习和创新性、技能性学习，加强公务人员专业化建设，更多用专业力量、专业方法解决发展中的问题。坚持学以致用，着力解决“不会为”问题、增强“善作为”本领，提高抓生态、抓发展、抓服务企业、抓基层治理等方面的能力。

　　打造服务型政府。进一步办好12345政务服务热线、96345市民服务热线，认真倾听、主动回应群众的呼声和关切，做到民有所呼、我有所应。坚持说到做到、取信于民，踏踏实实为群众办好每一件事，用我们的辛苦换得群众更多的轻松、快乐。加强政府权力清单、责任清单等清单的动态管理，深入推进政务服务流程优化再造，高质量履行公共服务、市场监管、社会管理、环境保护等职能职责，营建最优营商环境。

　　打造有为政府。发展是最大的担当，各项工作都要拉高标杆而不能降格以求，体现刚性而不能含糊其辞，精准管理而不能停留在“大概”“可能”“也许”。坚决摒弃“表态多、调门高”“行动少、落实差”，倡导勇于挑最重的担子、敢于啃最硬的骨头、善于接最烫的山芋，树立结果导向、实干导向。围绕推动全市域高质量发展，充分发挥县(市、区)能动性，进一步完善市域统筹发展机制，理顺市区共建机制。完善激励约束、容错纠错机制，为积极干事者撑腰鼓劲，对庸政懒政者严肃问责。改进督查考核，推进表格化、清单式、销号制动态管理。坚持重心向基层下移、力量向基层下沉、政策向基层聚焦、资源向基层倾斜，夯实基层基础。

　　打造法治政府。按照党中央、国务院和省委、省政府统一部署，有序推进机构改革，促进形成职责明确、依法行政的政府治理体系。深入学习贯彻宪法，推进市政府常务会议常态化学法，推动落实“谁执法谁普法”的普法责任制。研究制定加强法治政府建设的实施意见，协同做好地方立法工作，加快重点领域生态立法。强化政府规范性文件、重大决策、重大行政机关合同合法性审查，从源头上规范行政行为。深化行政复议体制改革，确保6月底前县(市、区)改革全部到位。制定政府法律顾问责任清单。严格规范公正文明执法，加强行政执法监督规范化建设。

　　打造清廉政府。严格落实全面从严治党主体责任，增强政治定力、纪律定力、道德定力、抵腐定力，高标准贯彻中央八项规定及实施细则精神和省委、市委实施办法，杜绝形式主义、官僚主义等“四风”突出问题隐形变异、反弹回潮，切实解决“中梗阻”问题。大兴调查研究之风，既要“身入”更要“心至”，察实情、体民忧、解难题。坚持过紧日子，力戒浮华，节俭办事。自觉接受人大依法监督、政协民主监督以及监察、司法、审计、群众、舆论监督，深入推进政务公开，营造政务生态的“绿水青山”。

　　各位代表！新时代是奋斗者的时代。让我们高举习近平新时代中国特色社会主义思想伟大旗帜，更加紧密地团结在以习近平同志为核心的党中央周围，在省委、省政府和市委的坚强领导下，凝聚起新时代奋进的伟大力量，高起点对标、高水平谋划、高标准建设、高要求落实，锐意进取、只争朝夕，为开辟“绿水青山就是金山银山”新境界而努力奋斗！
